# Development and Clinical Evaluation of Loop-Mediated Isothermal Amplification (LAMP) Assay for the Diagnosis of Human Visceral Leishmaniasis in Brazil

**DOI:** 10.1155/2019/8240784

**Published:** 2019-07-24

**Authors:** Daniel Moreira de Avelar, Débora Moreira Carvalho, Ana Rabello

**Affiliations:** Fundação Oswaldo Cruz, Instituto René Rachou, Pesquisa Clínica e Políticas Públicas em Doenças Infecciosas e Parasitárias, Belo Horizonte, MG, Brazil

## Abstract

Visceral leishmaniasis (VL) is considered a major public health concern in Brazil and several regions of the world. A recent advance in the diagnosis of infectious diseases was the development of loop-mediated isothermal amplification (LAMP). The aim of this study was to develop and evaluate a new LAMP assay for detection of K26 antigen-coding gene of* L. donovani *complex. A total of 219 blood samples of immunocompetent patients, including 114 VL cases and 105 non-VL cases, were analyzed for the diagnosis of VL in the present study. Diagnostic accuracy was calculated against a combination of parasitological and/or serological tests as a reference standard. The results were compared to those of kDNA* Leishmania-*PCR. The detection limit for the K26-Lamp assay was 1fg* L. infantum *purified DNA and 100 parasites/mL within 60 min of amplification time with visual detection for turbidity. The assay was specific for* L. donovani *complex. Sensitivity, specificity, and accuracy were 98.2%, 98.1%, and 98.2%, respectively, for K26-LAMP and 100%, 100%, and 100%, respectively, for kDNA* Leishmania*-PCR. Excellent agreement was observed between K26-LAMP and kDNA* Leishmania-*PCR assays (K = 0.96). A highly sensitive and specific LAMP assay targeting K26 antigen-coding gene of* L. donovani *complex was developed for diagnosis in peripheral blood samples of VL patients.

## 1. Introduction

Visceral leishmaniasis (VL) is a Neglected Tropical Disease (NTD) considered a significant public health problem in Brazil and several regions of the world [1]. Over 90% of VL human cases reported in the Americas occur in Brazil, where more than 41,000 cases were recorded between the years 2000 and 2011, with 3,322 deaths [2]. Despite the occurrence of a major technological breakthrough in recent decades, the laboratory diagnosis of VL still presents a challenge, likely to be an NTD, with little prospect of a commercial return on investment in research and development.

Parasitological diagnosis remains the gold standard in the diagnosis of VL. However, these methods require bone marrow or spleen aspiration, medical and invasive procedures, therefore inaccessible to most patients in endemic countries altogether [3]. Serological diagnosis is presented as an alternative to the parasitological techniques as it is less invasive and is facilitated by the substantial production of antibodies, during the clinical disease. Several assays using different antigens have been used for the detection of antileishmanial antibodies. Direct agglutination test (DAT), indirect fluorescent antibody test (IFAT), enzyme-linked immunosorbent assay (ELISA), and immunochromatographic tests (ICT) are currently used for VL diagnosis [3, 4]. Yet, in general, these methods present a number of limiting factors such as (i) the possibility of cross-reaction with other diseases, including Chagas disease and tegumentary leishmaniasis; (ii) persistence of positive antibody titers over long periods, even after treatment; (iii) low sensitivity in immunocompromised patients; (iv) expensive infrastructure and advanced technical skills required to conduct some these tests [5].

Over the 15 past years, several molecular techniques targeting various parasite genes have been developed for VL diagnosis. These have become increasingly relevant due to their remarkable accuracy, even using less invasive biological samples, as peripheral blood and serum [4]. Polymerase chain reaction (PCR)-based assays currently constitute the main molecular diagnostic approach: conventional PCR, nested-PCR, and quantitative real-time PCR. However, logistical, economic and technical reasons hinder the implementation of these molecular tools, particularly in high VL-burden countries where the majority of VL cases occur [3].

A recent advance in the diagnosis of infectious diseases was the development of technical loop-mediated isothermal amplification (LAMP). LAMP is a powerful innovative gene amplification technique emerging as a simple rapid diagnostic tool for early detection and identification of infectious diseases. The major advantages of using LAMP include its high specificity, robustness against inhibitors, fast amplification, and the fact that it does not require a thermal cycler to run the test since the reaction is performed at a single temperature [6]. The products can be detected visually using multiple parameters, including a magnesium pyrophosphate precipitate detectable by visual inspection or turbidimetry, metal ion detectors such as calcein, hydroxynaphthol blue, pico-green, malachite green, and using a DNA-intercalating dye, such as SYBR green [7].

The LAMP technique has been applied for the detection of* Leishmania donovani *complex in sand fly vectors [8], dogs [9], and humans [10–14]. The only targeted genes utilized in detection of DNA* Leishmania *were the kinetoplast minicircle genes (kDNA), 18S ribosomal DNA (rDNA), and ribosomal ITS1 [10, 11, 13]. The K26 is also known as hydrophilic surface protein B (HASPB) and it is used for the specific detection of* L. donovani *complex in PCR assays [15]. This study reports the development and evaluation of a LAMP assay for the detection of K26 antigen-coding gene of* L. donovani *complex in the blood samples of VL patients and compares the results to those of kDNA* Leishmania*-PCR, a test used in reference centers for VL diagnosis in Brazil, exhibiting high sensitivity and specificity. This is the first report of the LAMP assay to detect* L. infantum *DNA in human peripheral blood samples in Brazil.

## 2. Materials and Methods

### 2.1. Primer Design

**Figure 1:**
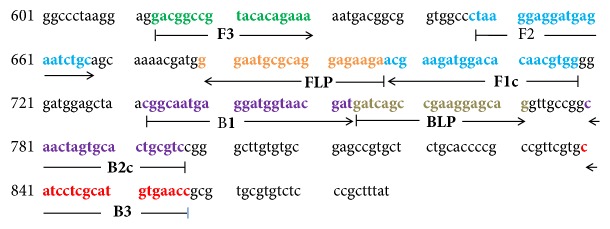
Location of LAMP primers in the* Leishmania donovani complex K26* gene. Internal nucleotide positions are within the* L. infantum* K26 gene (AF131228, positions 613-857, 879bp). F3, forward outer primer; B3, backward outer primer; F1c+F2, forward inner primer; B1+B2c, backward inner primer; FLP, forward loop primer; BLP, backward loop primer.

A set of six primers (K26-LAMP) complementary to the nucleotide sequence of* L. infantum* (GenBank accession no. AF131228) were designed using the PrimerExplorer v4 software (http://primerexplorer.jp/elamp4.0.0/index.html; Eiken Chemical Co., Ltd., Tokyo, Japan) according to the consensual criteria described by Notomi et al. [6]. The K26 gene of the* L. donovani *complex was chosen for amplification because it is able to characterize and distinguish strains of the* L. donovani *complex. K26-LAMP primers were specifically chosen to recognize six distinct regions on the target gene: external primers (F3 and B3), internal primers (FIP and BIP), and loop primers (Loop-F and Loop-B). The nucleotide sequence of the LAMP amplicon (Figure 1) and the LAMP primers (Table 1) were BLAST searched against the NCBI database to ensure their specificity (data not show).

### 2.2. Reference DNA and Optimization of Reaction Conditions for K26-LAMP

Genomic DNA was obtained from the following* Leishmania *reference strains:* L. *(*L*.)* infantum *(MHOM/BR/74/PP75),* L. *(*L*.)* donovani *(MHOM/ET/67/HU3),* L. (L.) amazonensis *(IFLA/BR/1967/PH-8),* L. (Viannia) braziliensis *(MHOM/BR/75/M2903), and* L. *(*V.*)* guyanensis *(MHOM/BR/1975/M4147). Samples of* Trypanosoma cruzi *(Y strains)*, Toxoplasma gondii *(ME49 strain), and* Schistosoma mansoni* (BH strain) were tested to ensure analytical specificity. DNA was extracted using QIAamp DNA Mini Kit (Qiagen Inc.) according to the manufacturer's instructions. The concentration of genomic DNAs was determined using a NanoDrop 1000 (Thermo Fischer Scientific Inc., Waltham, MA). All DNA samples were of sufficient quality, as indicated by their optimal 260/280 and 260/230 ratios. To estimate the analytical sensitivity of the LAMP assay, serially diluted* L. infantum *(MHOM/BR/74/PP75) DNA samples containing 1 ng to 0.1 fg were examined. To simulate infected blood, aliquots of peripheral blood samples obtained from healthy individual were added to the reference sample* L. (L.) infantum *(MHOM/BR/74/PP75) to obtain a concentration of 10,000 parasites/mL of blood. Serial tenfold dilutions, 10^6^-10^0^ parasites/mL) of* L. infantum *in human blood were made, and DNA was extracted using QIAamp DNA Mini Kit (Qiagen Inc.).

All LAMP primers used in this study were purified by high-performance liquid chromatography (HPLC) and purchased from Integrated DNA Technologies (Iowa, EUA). All the components of LAMP reaction master mix except betaine (Sigma-Aldrich) were from New England BioLabs (Ipswich, MA). An evaluation of the effects of different concentrations of MgSO_4_ (6 and 8 mM), betaine (0.8, 1.2, and 1.6 M), dNTPs (0.6, 1.0, and 1.4 *μ*M), the amplification temperature (61°, 63°, and 65°), and the reaction time (30, 45, 60, and 75 min) were carried out to optimize the K26-LAMP reaction. Amplification reactions were carried out using a water bath (Lindberg/Blue M, Thermo Fischer Scientific) and LAMP products were evaluated by visual inspection based on its generation of turbidity. For further confirmation, 4 *μ*L of the LAMP products was visualized after electrophoresis on a 6% polyacrylamide gel and silver stained. All tests were made in duplicate. Two technicians tested the same samples independently to confirm the reproducibility of LAMP results.

### 2.3. Calculating Sample Size, Clinical Samples, and Ethical Considerations

To determine the sensitivity, specificity, and reproducibility with a 95% confidence interval of the K26-LAMP, we first calculated the number of positive and negative blood samples necessary to assess these characteristics using the following equation [16]:

(1) $$\begin{array}{c} n\geq z^{2}xp\frac{\left(1–p\right)}{x^{2}} \end{array}$$

where* n* is number of positive or negative samples;* z,* 1.96; p, sensitivity (or specificity), and* x*^*2*^, confidence interval (CI). Considering a 95% CI and sensitivity and specificity of 0.95 a minimum of 73 positives and 73 negatives samples were required.

The Ethical Research Committee of the Research Centre René Rachou/FIOCRUZ approved this study (CAAE: 25393313.0.0000.5091). Blood samples which were recovered from the blood collection properly stored in freezer -80°C from the Clinical Research Laboratory at René Rachou Institute (CPqRR-FIOCRUZ) were used. To estimate the sensitivity, blood from 114 patients with clinical and laboratory diagnosis (direct microscopy examination of bone marrow aspirate or parasite culture, and/or the rK39 immunochromatographic rapid test) of VL were included. The specificity was evaluated using blood from 105 individuals who presented suggestive clinical presentation of VL (hepatosplenomegaly with fever) but negative results in parasitological diagnosis and rK39 immunochromatographic rapid test and a firm diagnosis of another disease. The noncases were diagnosed with various diseases, such as schistosomiasis mansoni, dengue, pancreatitis, cirrhosis, hepatitis, and syphilis. The kDNA* Leishmania-*PCR [17] was also performed in these samples and considered as a reference technique for accuracy. Diagnostic accuracy is expressed as a proportion of correctly classified subjects (true positives and true negatives) among all subjects.

### 2.4. DNA Extraction, K26-LAMP, kDNA* Leishmania-*PCR, and ACTB PCR

DNA from peripheral blood samples was extracted using QUIAamp DNA Mini (QIAGEN GmbH, Hilden, Germany) kit according to the manufacturer's instructions. Negative DNA extraction controls were performed for each experiment through the addition of all reagents except the sample. The yield was determined by absorbance at 260 nm in a spectrophotometer (NanoDrop ND-1000, Thermo Fischer Scientific, Wilmington, DE, USA). The A260/280 and 260/230 absorbance ratios were analyzed to verify the purity of the DNA obtained. To prevent cross-contamination, different sets of pipettes and different work areas were designated for template preparation, reaction mixture preparation, and DNA amplification.

The final optimized K26-LAMP assay conditions included incubation at 65°C for 75 min and then heated at 80°C for 5 min to terminate the reaction. The reaction mixture (25 *μ*L) consisted of 1.6 *μ*M each FIP and BIP primer, 0.8 *μ*M each Loop-F and Loop-B primer, 0.2 *μ*M each of F3 and B3, 8 U of* Bst*-WarmStart DNA polymerase; 1 mM deoxynucleoside triphosphates, 0.8 M betaine, 20 mM Tris-HCl (pH 8.8), 10 mM KCl, 10 mM [NH_4_]_2_ SO_4_, 8 mM MgSO_4_, and 1% Tween 20, and 3 *μ*L of template DNA. LAMP products were evaluated by visual inspection based on its generation of turbidity.

For kDNA* Leishmania*-PCR, the reaction was performed using the following* Leishmania *genus-specific primers: (i) a sense primer 150 (5′-GGGG/TAGGGGCGTTCTC/GCGAA-3′); (ii) an antisense primer 152 (3′-C/GC/GC/GA/TCTATA/TTTACACCAACCCC-5′) that anneals to the origin of replication on both strands of the minicircle to amplify the generic conserved sequence of the kDNA minicircles of 120 bp [17]. PCR was carried out in a total volume of 10 *μ*l containing 0.75 U of* Taq* DNA polymerase in 10 mM Tris-HCl, 50 mM KCl, 2 mM MgCl_2_ solution, 0.4 mM of each nucleotide (Promega Corp., Madison, WI, USA), 0.6 *μ*M of each primer (Integrated DNA Technologies, Iowa, EUA), and 1*μ*L of DNA sample. Thermocycling conditions were one step at 95°C for 5 min followed by 34 amplification cycles at 95°C for 30 seconds, 60 °C for 30 seconds, and 72 °C for 30 seconds. A final step of 72°C for 5 minutes was performed. Each PCR assay included negative (PCR mix without DNA and control for the process of DNA extraction) and positive (genomic DNA extracted from the reference strain of* L. *(*L*.)* infantum*, MHOM/BR/74/PP75) controls. All DNA-negative blood samples were assayed using specific primers for the human beta actin (*ACTB*) gene [18], and the presence of human DNA was confirmed. PCR products were visualized in 6% silver-stained polyacrylamide gels.

### 2.5. Statistical Analysis

The sensitivity, specificity, and diagnostic accuracy were calculated using a two-by-two contingency table with exact binomial at a 95% confidence interval (95% CI). The qui-square test was employed for comparison of sensitivities, specificities, and diagnostic accuracies presented by the K26-LAMP and kDNA* Leishmania*-PCR, considering a 5% significance level. The interobserver reproducibility of K26-LAMP assay results and the level of agreement between K26-LAMP and PCR assays were determined using the kappa index [19]: 1.00–0.81 excellent, 0.80–0.61 good, 0.60–0.41 moderate, 0.40–0.21 weak, and 0.20–0.0 negligible. The significance level was set at < 5% probability of *α* error.

## 3. Results

### 3.1. Optimization of K26-LAMP Assay

By using the primer sets selected, the results indicated that the Mg^2+^ concentration needs to be at least 8mM to give a positive reaction. Different concentrations of dNTPs and betaine were also tested in K26-LAMP reactions, and maximum amplification was established at 1mM and 0.8 M, respectively. No difference was found when the reaction temperature varied from 61° to 65°C. Although positive reactions could be obtained at 30 min, the product reached maximum amplification at 75 min.

### 3.2. Detection Limit and Specificity of the K26-LAMP Assay

**Figure 2:**
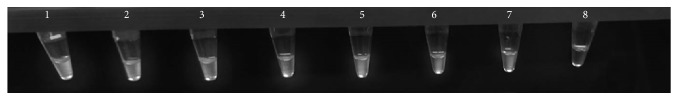
Analytical sensitivity of loop-mediated isothermal amplification (LAMP) for the detection of* Leishmania infantum *DNA. Turbidity increases in the positive reaction due to the generation of magnesium pyrophosphate. Tube 1, 1ng*; *Tube 2, 100pg; Tube 3, 10pg; Tube 4, 1pg; Tube 5, 100fg; Tube 6, 10fg; Tube 7, 1fg; Tube 8, 0.1fg.

**Figure 3:**
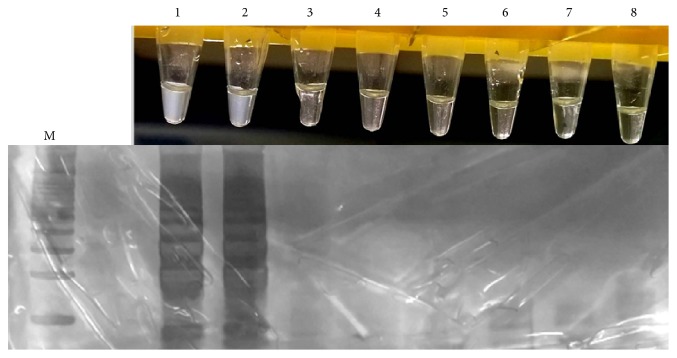
Specificity of the K26-LAMP assay for detection of* Leishmania donovani complex *DNA. Visual detection of the turbidity of the reactions solutions under natural light (top row); LAMP products studied by polyacrylamide gel electrophoresis (bottom row). Lane M, 100 bp DNA ladder; lane and tube 1,* L. donovani*; lane and tube 2,* L. infantum; *lane and tube 3,* L. amazonensis*; lane and tube 4,* L. braziliensis*; lane and tube 5,* L. guyanensis*; lane and tube 6,* Schistosoma mansoni*; lane and tube 7,* Trypanosoma cruzi*; lane and tube 8,* Toxoplasma gondii.*

The detection limit for the K26-Lamp assay was 1fg* L. infantum *purified DNA and 100 parasites/mL (Figure 2). In the evaluation of analytical specificity, the K26-LAMP assay showed positive results only for species from the* L. donovani *complex (*L. (L.). donovani* and* L. (L.) infantum*) and negative results for the other* Leishmania* species and parasite pathogens (Figure 3).

### 3.3. Diagnostic Accuracy of the K26-LAMP Assay and Comparison with kDNA* Leishmania*-PCR

Two hundred nineteen samples (114 VL cases and 105 noncases) were then tested using both methods. The K26-LAMP assay presented an overall sensitivity of 98.2% (95% CI: 93.8 to 99.8), specificity 98.1% (95% CI: 93.2 to 99.8), and 98.2% accuracy (95% CI: 95.4 to 99.5%). In comparison, the kDNA* Leishmania-*PCR assay presented 100% sensitivity (95% CI: 96.8 to 100%), 100% specificity (95% CI: 96.5 to 100%), and 100% accuracy (95% CI: 98.3 to 100). There was no statistical difference between sensitivity and specificity rates presented by K26-LAMP and kDNA* Leishmania*-PCR assays (p ≥ 0.05). Total agreement was observed in the test results between observers (K = 1.0) and excellent between K26-LAMP and kDNA* Leishmania-*PCR assays (K = 0.96; 95% CI: 0.93 to 0.99).  Table 2 lists the sensitivity, specificity, and accuracy rates observed for K26-LAMP and kDNA* Leishmania*-PCR assays.

## 4. Discussion

The effective control of VL involves reliable, rapid, and cost-effective diagnostic tools. Nucleic acid tests (NATs), such as PCR, have high precision rate for the laboratory diagnosis of VL [20]. However, these assays remain largely confined to reference laboratories due to the high cost of precision instrumentation and trained personnel. Among NATs, one alternative can be the LAMP assays that have been widely used for the detection of different pathogens [7, 21]. No thermal cycling is required and the method offers simplicity with very high sensitivity and specificity [21].

In the present study, a rapid and reliable diagnostic LAMP method that has high sensitivity and specificity for detecting* L. donovani complex *DNA in blood samples was established. The advantage of using whole blood samples is that they are obtained in a minimally invasive manner. Besides that, there was no statistically significant difference between the accuracy of molecular methods using whole blood samples in VL diagnosis compared with the use of bone marrow clinical samples [22]. The K26-LAMP assay was capable of detecting 100 parasites/ml blood and 1fg of* L. infantum *DNA purified from cultured promastigotes, which corresponds to 0.005 parasites per reaction (~200 fg total DNA for a single parasite) [23]. This detection limit was similar to that of other studies [9, 11, 13, 14, 24]. The K26-LAMP assay was specific for* L. donovani *complex. Contrary to the findings of Adams et al. [11] that found nonspecific amplification of* T. brucei *and* T. cruzi *DNA using primers for 18S region of* Leishmania*.

The evaluation of K26-LAMP assay exhibited a good performance, with a sensitivity of 98.2% and specificity of 98.1%. The diagnostic performance of K26-LAMP assay is consistent with previous reports of LAMP assays for VL diagnosis. The first LAMP test for the diagnosis of VL was developed by Takagi et al., using specific primers based on kDNA sequences for* L. donovani *[10]. Later, this same test was evaluated for the diagnosis of VL using blood [12, 23] and bone marrow aspirate samples from the patients [25], with sensitivity and specificity values ranging from 90.7-100% and 98.5-100%, respectively. One LAMP assay based in the use of a reverse transcriptase and 18S ribosomal RNA for the diagnosis of leishmaniasis showed sensitivity of 83% and specificity of 98% [11]. In Iran, other LAMP assay specific for* L. infantum *kDNA showed sensitivity of 93.6% and specificity of 100%, with similar results to those of nested PCR [13]. Recently, a novel pan-*Leishmania* LAMP assay was developed with primers based on 18S rDNA and kDNA. With VL suspects in Ethiopia, the sensitivity and specificity were 92% and 100% in whole blood [14].

Direct comparison of the K26-LAMP assay with diagnostic reference procedure kDNA* Leishmania*-PCR [17] for 219 clinical specimens (blood samples) indicated an excellent level of agreement between assays. The K26-LAMP assay was clinically less sensitive than the kDNA PCR, but there was no statistical difference between the two assays. One potential explanation for this difference is the large copy number (approximately 10,000 copies) of kDNA in the* Leishmania *cell making them ideal molecular target for detecting* Leishmania *[26]. In fact, PCR assays targeting minicircles kDNA conserved regions reached the lowest limit of detection (0.0125 parasites/ml of blood) [27, 28]. One additional procedural modification in K26-LAMP assay may also prove advantageous: the denaturation of the template DNA at 95°C for 5 min prior to the reaction has been reported to increase sensitivity [29].

Some kits, such as for the diagnosis of malaria and tuberculosis, are commercially available as Loopamp Assays, by Eiken Chemical Co. (Japan) (Available: https://www.human.de/products/molecular-dx/), and the latter was recently endorsed by the World Health Organization [30]. Recently, Eiken Chemical Co., FIND, and partners developed the Loopamp™* Leishmania* Detection Kit for the diagnosis of leishmaniasis. This kit was evaluated in Sudan for diagnosis of VL and obtained a sensitivity of 97.6% and specificity of 99.1%, using whole blood and buffy coat processed by a direct boil and spin method [31]. Although it was an excellent performance, further evaluation studies of this kit are necessary for different endemic regions of VL, including Brazil.

In this study, results of LAMP assay were interpreted by observing turbidity that reduces time and cost of postconventional PCR analysis [7, 12] and also eliminates the chance of contamination involved in gel electrophoresis [6]. To enhance the applicability of K26-LAMP assay in low resource clinical settings, simpler and user-friendlier DNA extraction method and a formulated ready-to-use reaction mixture should be available. Some studies have indicated the usefulness of using heat-treated samples as a template DNA source without the necessity of DNA extraction kits [12]. Also, LAMP assays are less susceptible than PCR to inhibitors that commonly occur in clinical specimens [6, 7]. In this paper a case-control diagnostic study was used and can lead to inflated estimates of accuracy [32, 33].

## 5. Conclusion

A highly sensitive and specific LAMP assay targeting K26 antigen-coding gene of* L. donovani *complex was developed for diagnosis in peripheral blood samples of VL patients. Further evaluation of K26-LAMP assay in prospective studies is necessary to confirm its clinical sensitivity, specificity, and predictive values compared with the main tests used in routine diagnosis of VL. Also, it is important to evaluate this assay in diagnosis of VL in HIV-infected patients.

## Figures and Tables

**Table 1:** K26-LAMP primers used in this study.

Primer^*∗*^	Sequence 5′ – 3′	bp
K26_F3	GACGGCCGTACACAGAAA	18
K26_B3	GGTTCACATGCGAGGATG	18
K26_FIP	CCACGTTGTGTCCATCTTCGT-CTAAGGAGGATGAGAATCTGC	42
K26_BIP	CGGCAATGAGGATGGTAACGAT-GACGCAGTGCACTAGTTG	40
K26_FLP	TCTTCTCCTGCGCATTCC	18
K26_BLP	GATCAGCCGAAGGAGCAG	18

^*∗*^ F3, forward outer primer; B3, backward outer primer; FIP, forward inner primer; BIP, backward inner primer; FLP, forward loop primer; BLP, backward loop primer.

**Table 2:** Sensitivity, specificity, and accuracy of K26-LAMP and kDNA PCR for detection of *L. donovani *complex DNA from blood samples. Comparison with parasitological diagnosis.

Participants	No of samples	K26-LAMP			kDNA-PCR		
		No of positives	Se % (95% CI)	Sp. % (95% CI)	No of positives	Se % (95% CI)	Sp % (95% CI)
Confirmed cases	114	112	98.2 (93.8-99.8)	-	114	100 (96.8-100)	-
Non-VL cases	105	02	-	98.1 (93.2-99.8)	0	-	100 (96.5-100)

## Data Availability

The data used to support the findings of this study are available from the corresponding author upon request.
